# Empirical Determination of Efficient Sensing Frequencies for Magnetometer-Based Continuous Human Contact Monitoring

**DOI:** 10.3390/s18051358

**Published:** 2018-04-27

**Authors:** Seungho Kuk, Junha Kim, Yongtae Park, Hyogon Kim

**Affiliations:** Department of Computer Science and Engineering, Korea University, Anam-Dong, Sungbuk-Gu, Seoul 02841, Korea; shkuk@korea.ac.kr (S.K.); zaqwsx3255@gmail.com (J.K.); ytpark@korea.ac.kr (Y.P.)

**Keywords:** smartphone magnetometer, coexistence detection, sampling frequency, energy consumption, disease epidemic contact tracing

## Abstract

The high linear correlation between the smartphone magnetometer readings in close proximity can be exploited for physical human contact detection, which could be useful for such applications as infectious disease contact tracing or social behavior monitoring. Alternative approaches using other capabilities in smartphones have aspects that do not fit well with the human contact detection. Using Wi-Fi or cellular fingerprints have larger localization errors than close human contact distances. Bluetooth beacons could reveal the identity of the transmitter, threatening the privacy of the user. Also, using sensors such as GPS does not work for indoor contacts. However, the magnetometer correlation check works best in human contact distances that matter in infectious disease transmissions or social interactions. The omni-present geomagnetism makes it work both indoors and outdoors, and the measured magnetometer values do not easily reveal the identity and the location of the smartphone. One issue with the magnetometer-based contact detection, however, is the energy consumption. Since the contacts can take place anytime, the magnetometer sensing and recording should be running continuously. Therefore, how we address the energy requirement for the extended and continuous operation can decide the viability of the whole idea. However, then, we note that almost all existing magnetometer-based applications such as indoor location and navigation have used high sensing frequencies, ranging from 10 Hz to 200 Hz. At these frequencies, we measure that the time to complete battery drain in a typical smartphone is shortened by three to twelve hours. The heavy toll raises the question as to whether the magnetometer-based contact detection can avoid such high sensing rates while not losing the contact detection accuracy. In order to answer the question, we conduct a measurement-based study using independently produced magnetometer traces from three different countries. Specifically, we gradually remove high frequency components in the traces, while observing the correlation changes. As a result, we find that the human coexistence detection indeed tends to be no less, if not more, effective at the sampling frequency of 1 Hz or even less. This is because unlike the other applications that require centimeter-level precision, the human contacts detected anywhere within a couple of meters are valid for our purpose. With the typical smartphone battery capacity and at the 1 Hz sensing, the battery consumption is well below an hour, which is smaller by more than two hours compared with 10 Hz sampling and by almost eleven hours compared with 200 Hz sampling. With other tasks running simultaneously on smartphones, the energy saving aspect will only become more critical. Therefore, we conclude that sensing the ambient magnetic field at 1 Hz is sufficient for the human contact monitoring purpose. We expect that this finding will have a significant practicability implication in the smartphone magnetometer-based contact monitoring applications in general.

## 1. Introduction

In just 17 years into the 21st century, we have already been through several novel pandemics such as SARS (2003), Swine Flu (2009), MERS (2012), Ebola (2014), and Zika (2015). Meanwhile, recent outbreaks have been fundamentally different from those of the past:—highly mobile populations [1] and the spread into densely populated cities are making them increasingly difficult to control [2]. On the brink of an infectious disease epidemic, the most urgent task is to trace those who made contacts with the confirmed case(s), in order to cut the chain of infection and prevent it from growing into a wider epidemic. However, the traditional contact tracing technique has been predominantly analog. Namely, contact graphs are constructed through interviews with confirmed cases, by asking who they remember they met and where they visited. This is a hugely costly and time consuming task. Worse yet, there is the issue of recall [3]. Therefore, there is a pressing need to develop a technology-based solution [2] to the contact tracing that overcomes these issues, so that we can cope with future infectious disease epidemics more efficiently and precisely.

### 1.1. Exploiting Smartphone Magnetometer for Contact Detection

**Figure 1:**
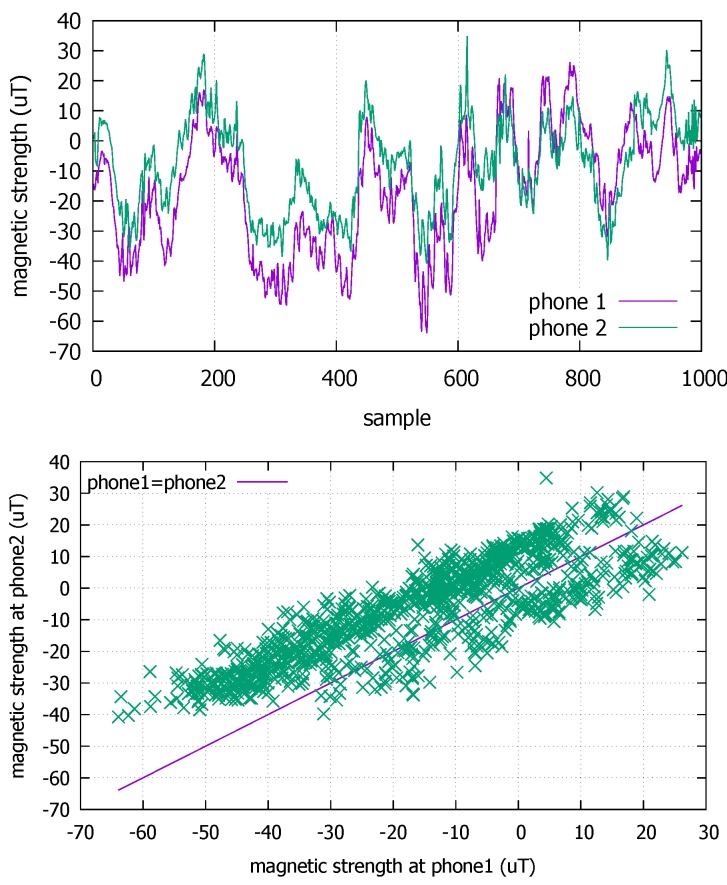
Magnetometer traces from two smartphones as the users walk together in a corridor (**top**); linear correlation is observed between the coexistent traces (**bottom**).

**Figure 2:**
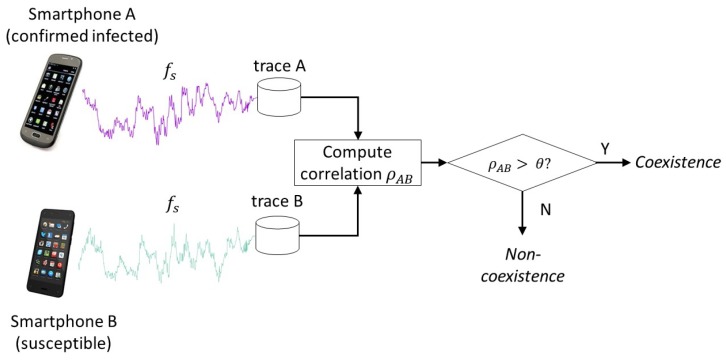
How the magnetometer traces could be used for contact detection.

When there are many potential contacts that an infected person cannot identify or recollect (as in public transports), a potent tool we can exploit is the mobile devices such as smartphones. The mobile-based epidemic monitoring is nothing but a logical development because only the mobile devices that move *with* people can keep up with the contacts they make. Indeed, there have been increasing number of proposals for smartphone-based contact tracing. The employed technologies range from similar Global Positioning System (GPS) positions [4], similar Wi-Fi fingerprints [5], Bluetooth peer discovery [6], and identical cells in mobile communication [7]. Unfortunately, they either provide position information too coarse to be used for infectious contact detection [8] (GPS, cellular/Wi-Fi fingerprinting), require infrastructure (cellular/Wi-Fi), cannot be used indoors (GPS), consumes high power (GPS) [9], or could compromise privacy (Bluetooth beacons). Some recent works [8,10], however, present a new possibility by demonstrating that magnetometer trace analysis can detect the coexistence in close proximity, works both indoors and outdoors, and offers better privacy protection by not revealing any identity. In particular, Kuk et al. [8] exploit that the magnetometer readings show high cross-correlation when two smartphones coexist in close distances that could enable disease transmission such as a couple of meters [11]. Figure 1 shows example traces generated by two phones held by the people walking side-by-side through a corridor in a university campus. We let the phones measure the ambient magnetic field strength in μT at the rate of 10 Hz. In the graph on top, the horizontal axis is the sample number of measured magnetometer values in the Z axis (perpendicular to the ground) and the vertical axis is the magnetic field strength. As predicted, these two 100-s time series do exhibit similar fluctuations. The fluctuations are the results of the magnetic distortions to the geomagnetic field by ferromagnetic materials such as steel doors, pillars, and rebars among others in the building the smartphone users are passing by. The synchronized fluctuations of the two magnetometer readings have a linear correlation, as shown by the graph at the bottom. Therefore, when each phone records such trace while the user moves around in daily life, we can let the users later check for possible contacts with an infected person using the linear correlation. For example, as in Figure 2, a susceptible user can check if her smartphone has a trace segment that computes a high correlation with an infected person’s trace that can be provided by the disease control authority. Note that the magnetometer traces themselves do not reveal the identity of the producer or the location where they were produced.

Please note that although we assume continuous measurement for the proposed system, the measurement duration is limited to a certain finite duration. As we mentioned at the beginning, the proposed system can be used for instance in the emergency situation while responding to an unfolding infectious disease epidemic or during a monitoring study in social sciences. Also note that one could imagine uploading the continuously measured data to the cloud, but it is not really necessary, because the trace comparison can be done by individual users. Automatically uploading the trace to a central repository will facilitate the contact tracing by the disease control authority, but it will be at the cost of heavy network traffic on the part of the user and of large storage and computation overhead on the part of the authority. Due to these facts, we do not strongly advocate the use of the cloud as the central trace repository.

### 1.2. Energy issue of Continuous Monitoring

Although the contact detection using smartphone magnetometer traces has many desirable properties as discussed above, the magnetometer has the energy consumption issue as any other sensors do [9]. Because people can encounter acquaintances and strangers anytime anywhere in everyday life, the magnetic field strength sensing should continuously run. With such continuous and extended sensing required for the contact tracing application, the issue is more acutely pronounced. To illustrate this point, Table 1 shows the actual energy consumption as we vary the sensing frequency. All high sensing frequencies in the table have been used in the existing magnetometer-based applications such as indoor location and navigation: 10 Hz [8,12], 25 Hz [13,14], 49.65 Hz [10], 50 Hz [15], 100 Hz [16,17], and 200 Hz [18]. Assuming a typical smartphone battery capacity of 2800 mAh (e.g., on Galaxy S5), our measurement using the Monsoon power monitor [19] shows that the operating duration of a fully charged device is approximately 24 h without sensing anything or running any application (`baseline’ in Table 1). When we run the magnetometer sensing in the background, it incurs additional energy cost and reduces the operating duration, or time-to-drain (TTD). The measurement shows that the sampling frequency significantly impacts the battery lasting time. For example, the sampling at 1 Hz reduces the TTD by less than an hour. However, 10 Hz sampling further reduces it by more than two hours, and 100 Hz, by more than seven hours. Such high sampling frequencies may well render the continuous background sensing impracticable. Worse yet, the baseline consumption in reality will be even higher because we normally run other applications as well. Then the TTD will be even smaller, making the energy reduction a more critical issue for the practicability of the continuous magnetometer sensing.

The natural question that arises from the above observation is whether we can avoid using the same high frequencies as are used for other magnetometer-based applications, in human coexistence detection. In this paper, we show that the answer is positive, and that lower sampling frequencies as low as 1 Hz do not hurt the human contact detection performance. The significance of the finding is that we can engineer the magnetometer-based solution to continuous contact monitoring to be more practical. Below, we corroborate our claim by investigating three independent smartphone magnetometer trace datasets. In doing so, we focus on the contacts made in the indoor contexts, because urban life is 90% indoors [20] and indoors is where most infection events take place.

### 1.3. Related Work

Magnetometer has been extensively used for indoor localization and tracking (but not much for coexistence detection). Researchers found that the indoor magnetic field is rich in spatial features (e.g., 1 m${}^{-1}$ to 0.01 m${}^{-1}$) due to various distortions by ferromagnetic materials such as reinforced concrete and metal doors [21], and easy to sense [17]. Moreover, the field is stable over long periods of time [14,16,22]. The richness and the stability of the magnetic field enables mapping (or `fingerprinting’), and magnetic map-based applications. The first application is indoor location. Chung et al. [23] showed that the geomagnetic anomaly can provide signatures for indoor locations that can be leveraged for sub-meter-level location accuracy. Frassl et al. [17] used magnetic maps with centimeter-level accuracy to localize a human or robot. Li et al. [14] discussed possible issues that can affect the precision and the feasibility of the fingerprinting approach for indoor location. Angermann et al. [16] found that the use of all three field components provides good resolution of ambiguities in a small indoor area. Carrillo et al. [13] used the three components of the measured magnetic field by smartphone magnetometers instead of just the intensity to improve accuracy.

The second application is navigation. Brzozowski and Kazmierczak [24] discussed ways of recording, visualizing, and mapping local magnetic field changes in 3D that can be used as a support for indoor navigation systems for unmanned aerial vehicles (UAVs). Riehle et al. [18] considered a leader-follower style navigation application for visually impaired people where there is time gap between traversals, without relying on expensive indoor magnetic fingerprinting. A 200 Hz magnetometer sensing output from a body-worn inertial measurement unit (IMU) was time-tagged, and used as the leader trace. A follower could compare its own magnetometer trace and the leader’s to determine if the follower reached a waypoint and if the follower went off-route.

The third application also does not require fingerprinting, and it is of our interest in this paper—coexistence detection. Nguyen et al. [10] used only smartphone magnetometers to detect co-location of passengers in public transport. They exploited the fact that the passengers share the trajectory between at least two consecutive stations, and the magnetometer traces exhibit high similarity, which was measured by the distance in Derivative Dynamic Time Warping (DDTW). Kuk et al. [8] showed that even in outdoors the magnetometer traces can be compared to detect contacts within a few meters where the current GPS can have an order-of-magnitude larger errors. It showed that two closely located smartphones generate highly correlated magnetometer traces, which can be exploited to detect coexistence. These works also employed relatively high sampling frequencies (10 Hz [8] and 49.65 Hz [10]) as in the fingerprinting for location/navigation, but none investigated whether such high frequencies are absolutely necessary for human coexistence detection. In the rest of the paper, we pursue this issue.

### 1.4. Summary of Our Work

The existing magnetometer-based studies that used high sensing frequencies [8,10,12,13,14,15,16,17,18] implicitly assume that the higher the frequency, the better the precision. Indeed, some applications such as indoor location and navigation may need a centimeter-level resolution for the safest movements of robots and UAVs. However, for the human contact detection, we do not need such high precision. For instance, more rough distance estimates (e.g., a couple of meters [11]) may qualify for tracing possible infectious disease transmissions. Such loose requirement is also likely to apply to other human contact-based applications such as social interactions monitoring [25,26]. Therefore, in this paper, we aim to find appropriate magnetometer sensing frequencies that better fit the human contact detection applications.

Obviously, our investigation would be much easier if we had a general model of the indoor geomagnetic anomalies to identify the most effective sensing frequencies. Unfortunately, it would be a major undertaking to generalize the indoor magnetic distortion by various ferromagnetic objects even in one given building ([21], Chapter 6). Worse yet, every building can be different from others in terms of the structure and the ferromagnetic objects situated in it. Indeed, most of the aforementioned related works rely on measurements due to this difficulty, so we also take the measurement-driven approach to the problem. Specifically, we use independently produced indoor magnetic trace datasets from three different countries including our own, and determine the most desirable frequencies based on these trace data.

The principal finding of our investigation is that the coexistence feature is comparably (if not more) reliably detected at the sampling frequencies of 1 Hz or even less, in all three independent datasets. Not having to use the high sensing frequencies as used in other magnetometer-based applications is a good news for the energy consumption aspect of the magnetometer-based human contact detection. It will promote the practicability of the concept of magnetometer-based human contact monitoring that constantly runs in the background.

## 2. Materials and Methods

### 2.1. Indoor Magnetometer Traces

For our trace-based study, we use three independent sets of indoor magnetometer traces produced in three different countries. The first is what we collected in the Korea University campus in Seoul, Korea. On top of that, we use two publicly available magnetometer traces. To the best of our knowledge, these are among the few that are publicly available [12], and at the same time have the data volume large enough for our experiments. For instance, there is one more public trace data from Torres-Sospedra et al. [27], but the recorded trajectories are too short for our purpose. Among the two public datasets used in this paper, the first is from the Italian National Research Council building in Pisa [12] (http://wnet.isti.cnr.it/software/Ipin2016Dataset.html). The second public dataset we use is from three buildings in the University of Illinois, Urbana-Champaign [15] (http://bretl.csl.illinois.edu/magpie). For convenience, we will call these the KU, the Pisa, and the Illinois dataset, respectively.

#### 2.1.1. Korea University Campus Traces

**Figure 3:**
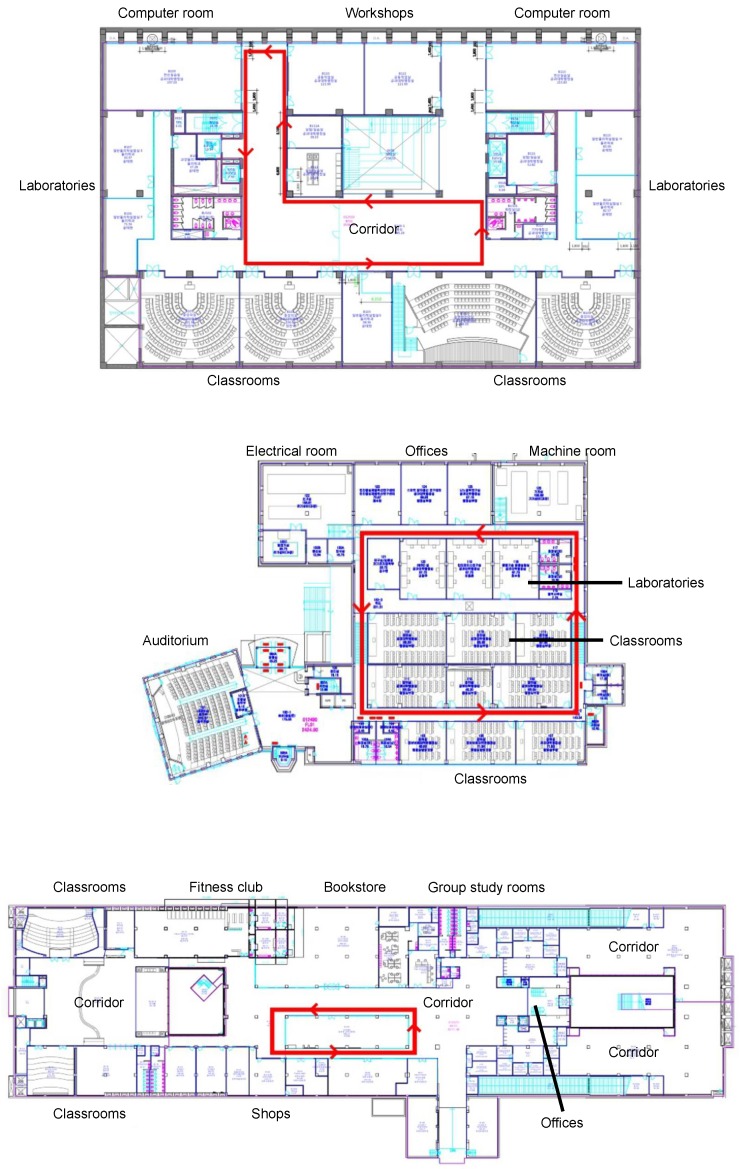
Trace generation environment in Korea University: Engineering College Building (**top**), Convergence Technology Hall (**middle**), and Campus Square (**bottom**).

In the Korea University measurements, two/three people with smartphones walked through three different campus buildings. The smartphones measured the magnetic field strengths in three orthogonal smartphone axes (X, Y, Z) at the base sampling frequency of 10 Hz. The buildings, Engineering College Building (ECB), Convergence Technology Hall (CTH), and Campus Square (CSQ), have the structures as shown in Figure 3. The first two are mostly composed of laboratories and classrooms. The third is an indoor mall with various shops, college offices, and lecture halls. Figure 3 also shows the walking paths used for trace collection (thick arrows). We used three different phones, LG G4, Galaxy S5, and Galaxy S6, each held by a walking person. We let the smartphone holders walk approximately at the `preferred’ walking speed [28]. It is known that people prefer to walk at approximately 1.4 m/s (or 5.0 km/h) irrespective of cultures, as they find slower or faster speeds uncomfortable. The smartphone holders walked side by side, keeping each other at arm’s length to simulate the typical personal gap [29]. For each building, we repeated the measurements over the same walking path five times. Each trace is two to three minutes long.

As for our measurement software, we wrote and installed a magnetometer sensing app on the Android smartphones, and synchronized their sensing activity through the Network Time Protocol (NTP) for later comparison of their magnetometer traces. Also, the sample values were translated to the absolute coordinate (i.e., North, East, etc.) using the Android getRotationMatrix() method to cope with the phone attitude changes. A desirable property of the magnetism is that it has absolute reference directions such as the East and the North. Smartphones can change attitudes freely, but the Android method lets us readily compare the traces from different phones independent of the attitude changes that take place in the compared smartphones. When computing the correlation between the traces, we took the one with the *maximum* correlation from among all three axes lest we miss even a single contact event.

#### 2.1.2. Italian National Research Council traces

**Figure 4:**
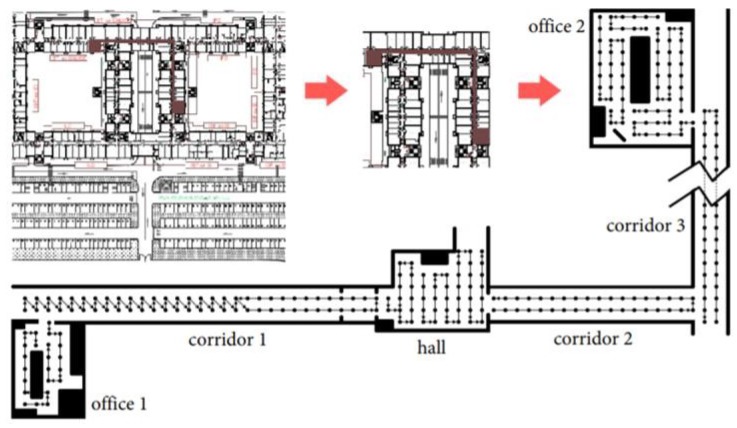
Trace generation environment for the Pisa traces [12].

**Figure 5:**
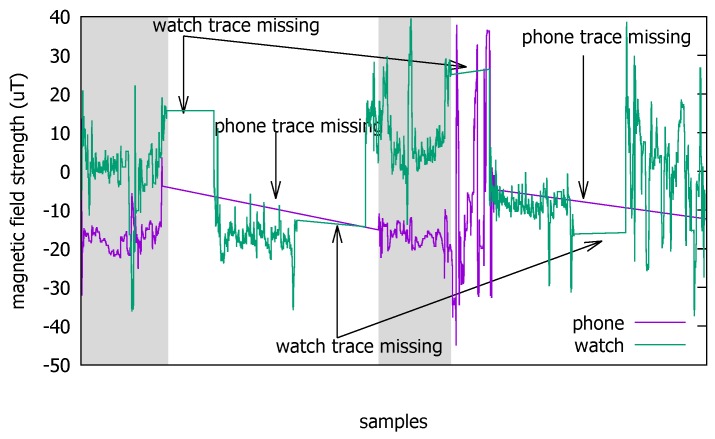
Magnetometer traces from the two mobile devices partially overlap over certain time periods in the Pisa traces.

The Pisa dataset consists of two trajectories, each of which contains multiple non-consecutive traces. In total, there are 36,795 samples of accelerometer, magnetometer, and gyroscope measurements collected in a 185 m${}^{2}$ indoor space in two measurement campaigns in the Italian National Research Council in Pisa [12] (Figure 4). The trace generation environment is composed of four hallways with fire doors and three rooms. The samples were taken at 10 Hz on a smartphone and a smartwatch worn by two users moving together. The movement speed was approximately 0.6 m/s. The reference time used by the smartwatch was synchronized with that of the smartphone before starting each data acquisition campaign. From the Pisa data, we only use the portions that have *both* the smartphone and the smartwatch measurement samples present because there are time intervals in which either device was not recording the sensed values. For example, the shaded sections in Figure 5 are what we use to test coexistence.

#### 2.1.3. University of Illinois Campus Traces

**Figure 6:**
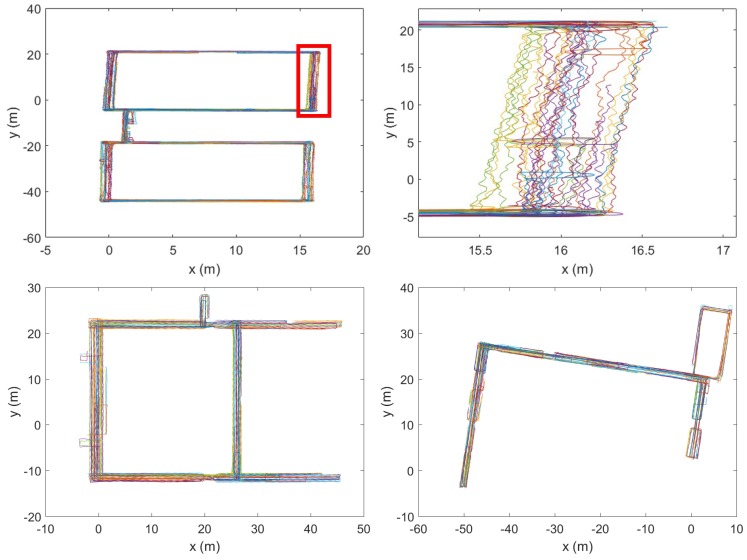
Ground-truth trajectories in three buildings: CSL (**top left**), part of CSL magnified (**top right**), Loomis (**bottom left**), and Talbot (**bottom right**) in the Illinois traces [15].

In the Illinois dataset [15], there are two types of data: unmanned ground vehicle (UGV) and walk (WLK). The former was obtained from a mobile robot, and the latter was from a walking human. We use the WLK type because our interest is in human coexistence detection. For the WLK dataset generation, a person carried the test phone in his hand to collect the magnetometer data at the sampling frequency of 50 Hz. There are a total of 299 trajectories, obtained by moving in the three buildings (CSL, Loomis, and Talbot) as shown in Figure 6. In the figure, all geometric shapes are the results of plotting the ground-truth position traces. (The explicit structural information about the three buildings such as floor plans is not provided in the Illinois dataset.) Note that the 299 trajectories partially overlap in space. For instance, the top right shows the magnified trace sections in the red highlighted box in the CSL trajectories on the left. For brevity, we do not show the magnified traces in the Loomis and the Talbot trajectories. On average, each trajectory is approximately 90 s and 101 m long. Since the Illinois traces were generated by a single smartphone (i.e., no prearranged coexistence condition), we artificially overlap two arbitrarily chosen traces and test if they are from the same walking path. The trace overlapping test is possible because the Illinois traces have the ground-truth position information coded together with the magnetometer readings (Figure 6). Among the 299 traces, there are a few that were generated from the same trajectory, and others from different parts of the buildings. Among them, we choose the ones that have overlapping coordinates moving in the same direction for more than 20 s for coexistence detection tests.

### 2.2. Finding Desirable Sampling Frequencies

#### 2.2.1. Computing Correlation

In order to determine the coexistence of two phone users, we compare two time-synchronized magnetometer traces for the KU and the Pisa traces. For the Illinois traces, we instead use the ground-truth positions to align the traces for coexistence detection test. Among the three axes in which the magnetometer measurements are produced, we consistently use the *Z* axis (perpendicular to the ground) for the correlation computation in case of the KU traces. For the Pisa and the Illinois traces, we manually inspected them and chose for each experiment an axis in which the true coexistent phones show the highest correlation. In a real implementation of the proposed system, one could compare the correlation coefficients computed in the three axes and pick the highest one for the coexistence decision. Because coexistent magnetometers produce a linear correlation (see Figure 2), we compute the Pearson cross-correlation coefficient between the time-matching sets of *N* consecutive measurements from the phones/watch while sliding over the entire compared magnetometer traces with stride 1. A Pearson correlation coefficient between the two traces from phones A and B for the window of size *N* starting from the $k^{\mathrm{th}}$ sample is computed as follows:

$$\rho_{k}\left(A,B\right)=\frac{1}{N-1}\sum_{i=0}^{N-1}\left(\frac{A_{k+i}-\mu_{A}}{\sigma_{A}}\right)\left(\frac{B_{k+i}-\mu_{B}}{\sigma_{B}}\right),$$

where $A_{k+i}$ and $B_{k+i}$ are ${\left(k+i\right)}^{\mathrm{th}}$ individual magnetometer readings from phones *A* and *B*, respectively. μ and σ are the mean and the standard deviation of the measured magnetometer strengths in the two phone’s compared traces. Note that these values are available at the time of trace comparison as the comparison is part of the post-mortem analysis (see Figure 2). Given *L* as the total length of the compared traces (i.e., the number of magnetometer readings in the trace), we obtain L−N+1 correlation coefficients. Among these, we take the maximum as the final measure of coexistence. Namely,

$$\rho_{max}\left(A,B\right)=\max_{k=1}^{L-N+1}\rho_{k}\left(A,B\right).$$

One may consider the two phones A and B made a contact if $\rho_{max}\left(A,B\right)>\theta$, where θ can be a predefined decision threshold [8]. In this paper, we set the sliding window *N* to span 20 s (i.e., 200 samples at 10 Hz), assuming that less than 20 s is too short to enable infectious disease transmission. The parameter *N* may need to be adjusted depending on the given disease, however, e.g., if even smaller contact time matters. Note that the type of contact we aim to detect in this paper is coexistence, or `trajectory bundle’ [30]. It is because this `same-place-same-time’ (SPST) contact type is more common in infectious disease transmissions than the `same-place-different-time’ (SPDT) type [31] (not to mention social contacts). Since the smartphone users are assumed to stay/move together in this type of contact, we do not need to align the traces for the time gap and the moving speed differences by using such schemes as Dynamic Time Window (DTW). Furthermore, since we use the maximum correlation coefficient as the decision input, only the strongly positive correlation coefficients signal the trajectory bundle. All other cases are classified as non-coexistence. Even strong negative correlation coefficients (i.e., close to −1), if any, are considered haphazard and irrelevant to the coexistence condition.

#### 2.2.2. Evaluating Frequency Contributions Using Low-Pass Filtering

The strategy we take for energy saving in this paper is to find the lowest sampling frequency for the smartphone magnetometer that do not hurt the coexistence detection performance. We utilize the three independent datasets for this purpose. Namely, we identify the desirable sampling frequency by exploring them, and propose it as a guideline for the indoor coexistence detection using the smartphone magnetometer. Specifically, we proceed as follows with the trace data. If $f_{s}$ is the original sampling frequency of the trace, the highest frequency component in the signal is $f_{s}/2$ (Nyquist-Shannon sampling theorem). As mentioned above, $f_{s}=10$ Hz in the KU and the Pisa traces, and 50 Hz in the Illinois traces. To these traces, we apply the low-pass filter (LPF). Starting from the highest frequency components in a given trace, we chip away at them by gradually lowering $f_{c}$ from $f_{s}/2$ to 0.1 Hz, while computing the correlation coefficients with the other identically lowpass-filtered trace. Then we observe what was the impact of the removed frequency component to the original correlation coefficient. If there is impact below a certain frequency cutoff $f_{c}^{*}$, we cannot lower the sampling frequency to below $2f_{c}^{*}$, or we run the risk of altering the decision that we would make with the original high sampling frequency. Specifically, we should make sure that the lowered cutoff cannot cause a false negative decision for coexistence or a false positive decision for non-coexistence. As long as these conditions are met, we can let $2f_{c}^{*}$ serve as the lower bound of the sampling frequency that we should actually use. As for the low-pass filter applied to the trace datasets, we used the butter() function in MATLAB to design a filter of order 5 and then the filtfilt() function to actually perform the filtering.

#### 2.2.3. Avoiding Over-Filtering

**Figure 7:**
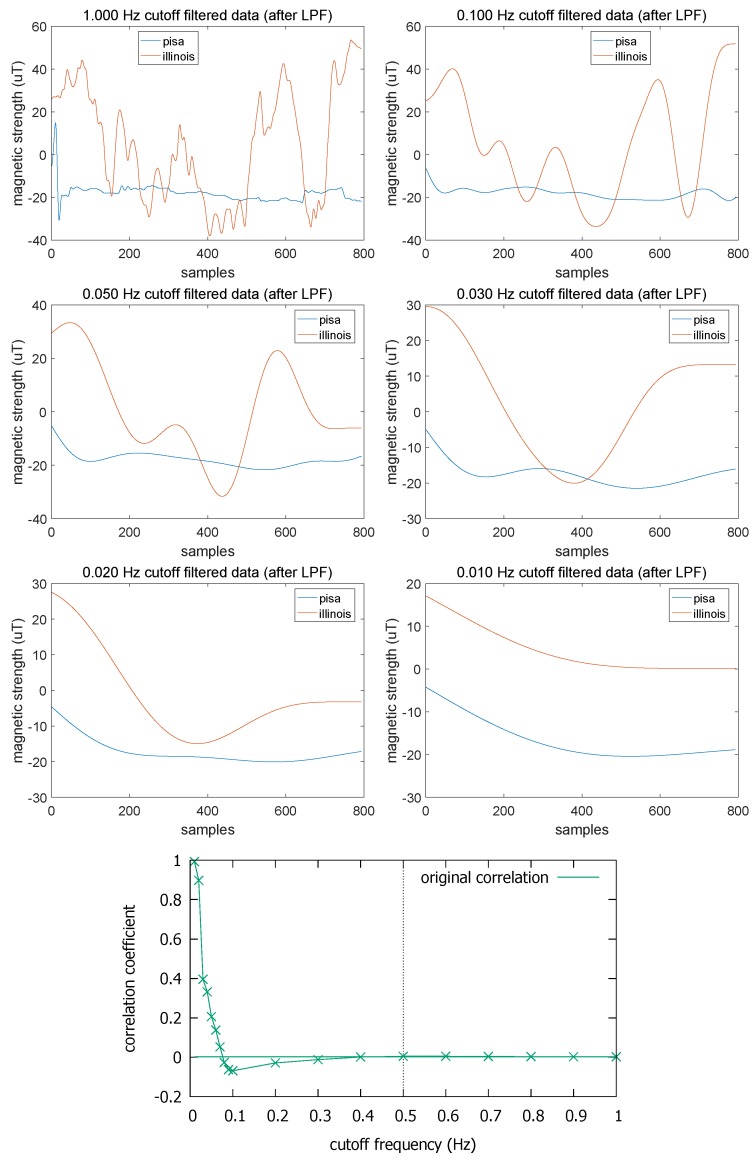
Overdoing the filtering increases the false positive rate: an example of Pisa-Illinois trace pair.

One caveat with LPF is that the more we lower $f_{c}$, the more frequency features we lose. In the extreme ($f_{c}\to 0$), all magnetometer fluctuations are flattened, and we will not be able to differentiate trajectories even if they come from totally different places. Ultimately, it would increase the false positive rate (FPR). Figure 7 illustrates what would happen if we excessively lowered $f_{c}$. Here, we picked one trace from the Illinois dataset (downsampled to 10 Hz) and the other from the Pisa dataset. Because there is no spatial correlation between these traces whatsoever, the correlation coefficient before LPF is 0.002 (Figure 7(bottom)). Then we gradually lower $f_{c}$ down to 0.01 Hz. As we can observe from the figure, the correlation coefficient naturally approaches 1 as $f_{c}\to 0$ because LPF eventually leaves only the DC component. As a minimum safeguard, we stop the LPF at $f_{c}=0.1$ Hz as mentioned above. For more detail on the actual cutoff point, we discuss it in the next section.

## 3. Results

In this section, we present the results of applying LPF to the three independent trace datasets to find the lowest `safe’ cutoff frequency $f_{c}^{*}$. Namely, we make sure that it does not alter the coexistence decision that the original trace would enable us with. Specifically, we test the impact of the lowered cutoff in the true positive and the true negative situations. Then after identifying the $f_{c}^{*}$, we actually use the recommended sampling frequency $f_{s}^{*}=2f_{c}^{*}$ natively in our trace collection, and see if it works as well as with the higher frequency sampled traces.

### 3.1. Impacts of Lowering Sampling Frequency in True Positive Situations

#### 3.1.1. KU Traces

**Figure 8:**
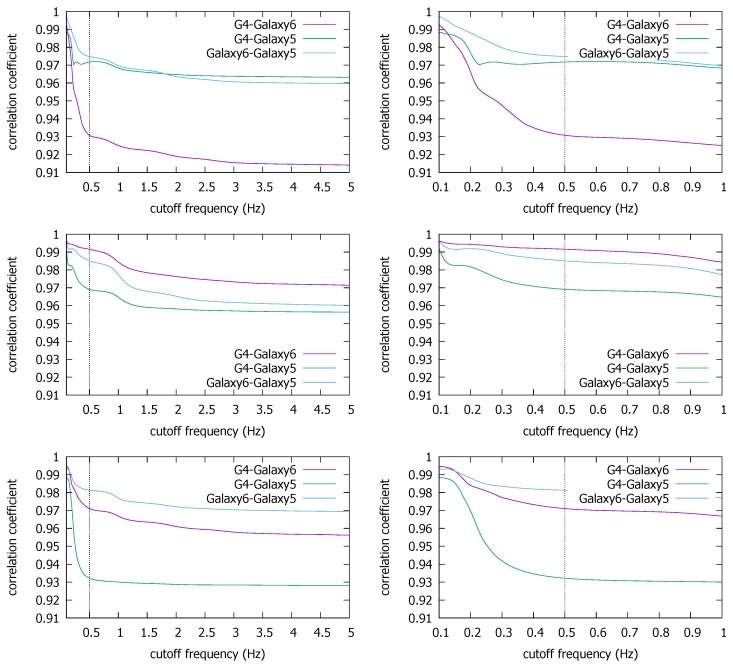
Average $\rho_{max}$ for coexistent traces in the Korea University dataset: ENB (**top**), CTH (**middle**), CSQ (**bottom**).

Figure 8 shows the LPF results for the coexistent KU traces where the cross correlation $\rho_{max}$ between two coexisting smartphones is drawn as a function of $f_{c}$. We plot the average over the five repetitions performed in each building (ENG, CTH, and CSQ), and in the right column, we magnify the low frequency region below $f_{c}=1$ Hz. What surprises us the most is that the higher frequency components seem to deteriorate the cross correlation rather than add to it. Namely, the correlation generally increases as we decrease the cutoff frequency. It implies that the high frequency components in the magnetometer readings contain more noise than signal, as far as the coexistence detection is concerned. This is a good news for energy saving because it means using only low frequency components would not significantly hurt the detection performance in the true coexistence situation.

We notice that there are trace pairs from the ENB and CSQ buildings whose correlation steeply increases as we lower $f_{c}$ below 0.5 Hz. Because it may have come from the loss of information as we observed in Figure 7, we conservatively estimate $f_{c}^{*}=0.5$ Hz (or $f_{s}^{*}=1$ Hz), which we will use below against the two other datasets as well. We also observe that there is little risk of causing false negative decisions by lowering the $f_{c}$ to 0.5 Hz, since it consistently *increases* the correlation. It contrasts with the uncorrelated traces case in Figure 7 where the correlation does not move as we decrease $f_{c}$ (until it begins the unwarranted surge due to information loss). There, it is natural because no coexistence condition is there to synchronize common frequencies. Essentially, everything is noise.

#### 3.1.2. Pisa Traces

**Figure 9:**
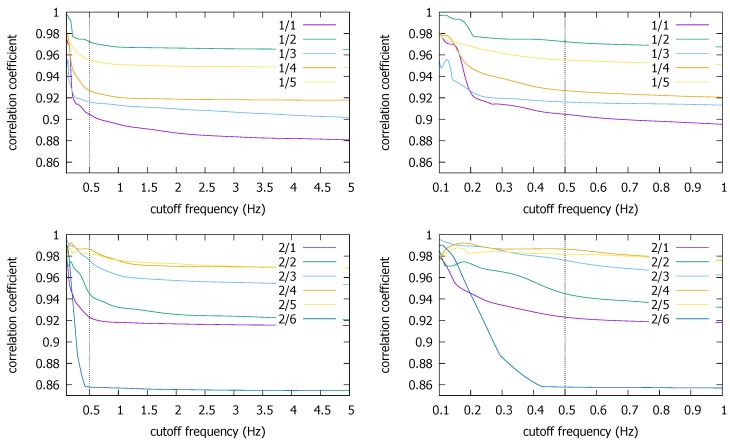
$\rho_{max}$ for coexistent traces in the Pisa dataset: Campaign 1 (**top**), Campaign 2 (**bottom**).

**Figure 10:**
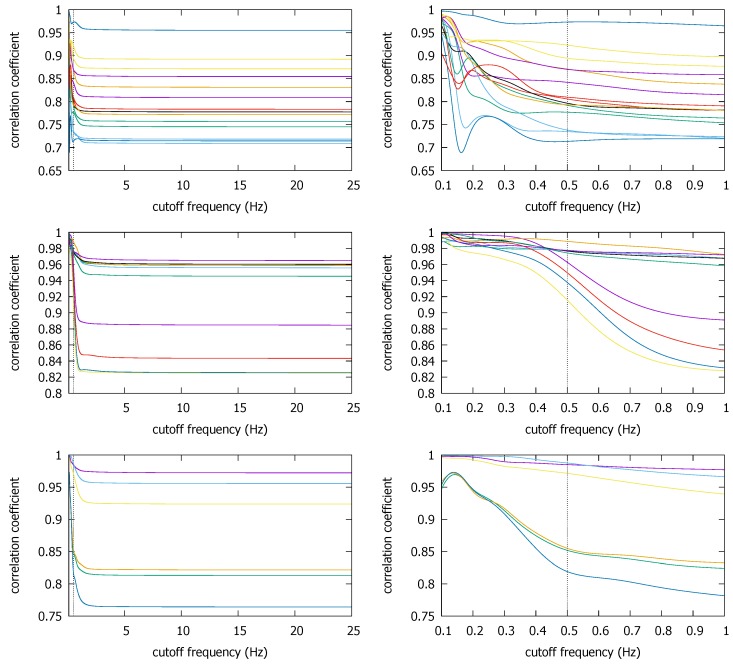
$\rho_{max}$ for coexistent traces in the Illinois dataset: CSL (**top**), Loomis (**middle**), Talbot (**bottom**).

The Pisa and the Illinois traces also seem to support our finding that lowering $f_{c}$ generally increases the correlation coefficient for the true coexistent situation (Figure 9 and Figure 10). Recollect there are two trajectories from two measurement campaigns in the Pisa dataset [12]. The graph on top in Figure 9 is for the first campaign, the bottom the second. In the former, there are five trace segments that have both the smartphone and the smartwatch data while the devices move together. In the latter, there are six. (In the graphs, `a/b’ refers to b^th^ trace from Campaign a.) We observe that $\rho_{max}$ values are both high for the coexistence in the two campaigns, close to 0.9. Again, as we have seen in the KU dataset, the $\rho_{max}$ value tends to increase toward to very high values as we lower $f_{c}$. In particular, the increase in the correlation is consistently observed as we decrease $f_{c}$ down to our suggested cutoff value of 0.5 Hz. Any fluctuations are much below the cutoff. Again, since the reduction of $f_{c}$ down to 0.5 Hz only increases the correlation, there is little risk of causing the false negative decision.

#### 3.1.3. Illinois Traces

In the Illinois traces, we select several overlapping traces (see Section 2.1.3) for each building. First, for CSL, we pick six overlapping traces #3, #9, #15, #16, #17, and #97, according to the order of the traces as contained in the published dataset [15]. For Loomis (#1, #3, #4, #5, #85) and Talbot (#1, #3, #6, #47), we similarly pick the traces that have overlapped positions for more than 20 s. For these two buildings, the traces that meet these spatial and temporal requirements are rarer, hence the smaller number of traces. Figure 10 shows that for all coexistent pairs, $\rho_{max}$ remains quite stable over most frequency ranges, and very slowly increases as we decrease $f_{c}$ (labels for the pairs are omitted to avoid clutter). Notice how little the high frequencies contribute to $\rho_{max}$. Only at very low $f_{c}$ do visible disturbances appear. Although a very few slightly decreasing correlations are observable around 0.5 Hz, the cutoff frequency reduction down to this point generally increases the correlation coefficients. In particular, for the Loomis and the Talbot, the increases become more significant towards 0.5 Hz. This surge may have originated from the information loss. Even if it is an error, however, it would not cause the false negative decision because the coefficient is moving in the increasing direction. Thus the cutoff at $f_{c}^{*}=0.5$ will be acceptable as long as does not cause the false positive decision for non-coexistent situations, which we discuss it in the next subsection.

The three datasets we use in this section are not only from different locations but were collected using different methods. For one, the Pisa and the Illinois traces do not use absolute coordinates, so the sample values may have been affected by phone attitude changes. Moreover, the Pisa trace has relatively short durations, and the Illinois traces were not generated in true coexistent conditions. Despite these variabilities, a common feature that we observe through the investigation in this section is that in all three datasets, the correlation coefficient generally increases with lower cutoff frequencies in the LPF. It means that we can use lower sampling frequencies (e.g., 1 Hz) on the smartphone magnetometer already in the trace collection step, without too much concern about losing contact-signaling frequency components from the trace.

### 3.2. Impacts of Lowering Sampling Frequencies in True Negative Situations

**Figure 11:**
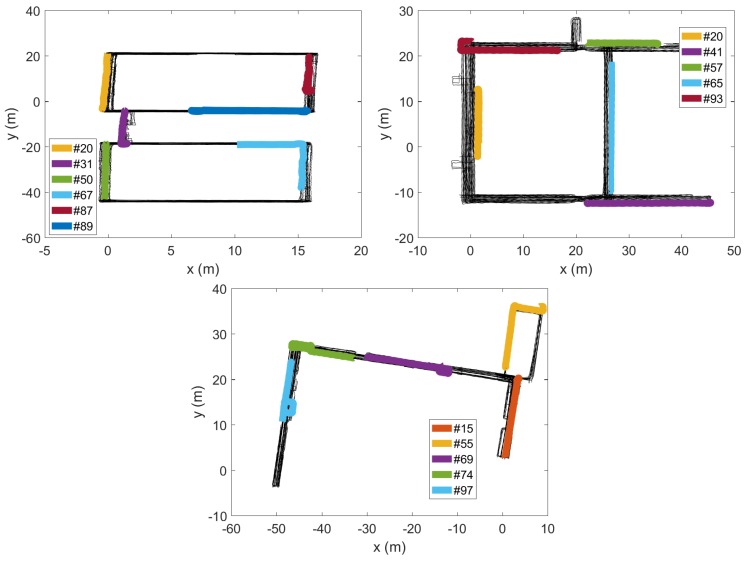
Non-coexistent traces selected from CSL (**top left**), Loomis (**top right**), and Talbot (**bottom**).

Having seen that there is little chance that the cutoff (hence sampling) frequency reduction will cause false negatives, one may wonder if it can cause false positive decisions on non-coexistent trace pairs. Between the traces produced from different countries, a false positive decision is less likely due to completely different trace collection environments (e.g., as we saw in Figure 7), so here we focus on intra-site trace pairs that were generated from different buildings/campaigns in the same site. Compared with the relatively small number of coexistent traces in the three datasets, there are numerous non-coexistent combinations we can create from them. In the Illinois dataset, we pick the non-coexistent traces for each building as shown in Figure 11.

**Figure 12:**
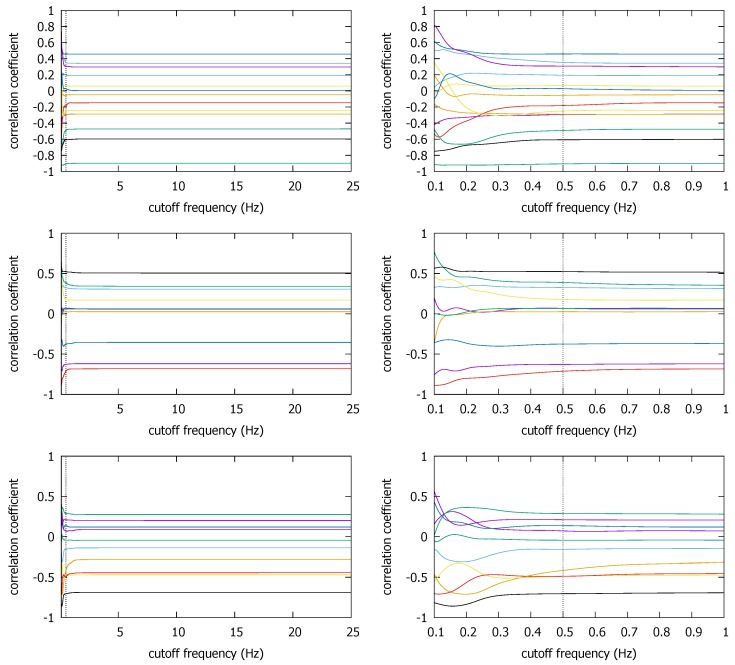
$\rho_{max}$ for non-coexistent trajectories from the three buildings in the Illinois dataset; CSL (**top**), Loomis (**middle**), and Talbot (**bottom**).

Figure 12 shows that the $\rho_{max}$ values for these non-coexistent pairs remain static until we reduce $f_{c}$ to much below 1 Hz. The figure also confirms our early finding that we cannot push down $f_{c}$ to too low a value such as 0.1 Hz, as some non-coexistent trace pairs can end up with a high correlation coefficient. For instance, one non-coexistent pair in the CSL case achieves $\rho_{max}>0.8$ at $f_{c}=0.1$ Hz. Depending on the decision threshold θ, it could cause a false positive decision. However, at the earlier suggested value of $f_{c}^{*}=0.5$ Hz, $\rho_{max}$ at the frequency is similar to that at the original sampling rate of 50 Hz (or $f_{c}=25$ Hz). Namely, as long as we do not reduce the sampling rate to much below 0.5 Hz, or set θ dangerously close to 0.5 (which is absurdly lower than the lowest correlation coefficient of approximately 0.7 at $f_{c}^{*}=0.5$ Hz in the coexistent cases—see Figure 10), the false positives can be avoided.

**Figure 13:**
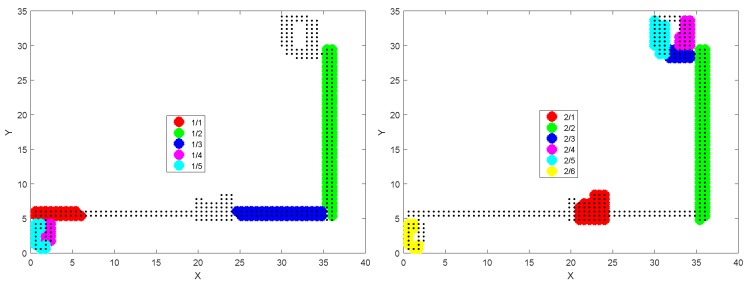
The Pisa traces from Campaign 1 (**lef**t) and Campaign 2 (**right**).

**Figure 14:**
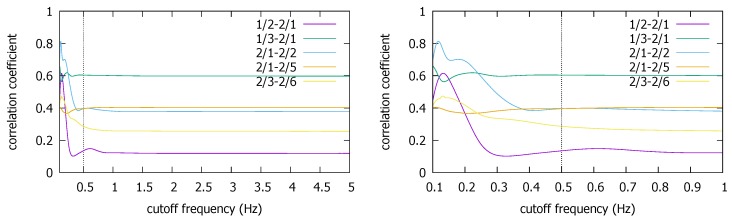
$\rho_{max}$ for non-coexistent trace pairs from the Pisa dataset.

We also observe similar results in the Pisa and the Korea University datasets. In the Pisa dataset, the trace segments are situated as in Figure 13. By using only the traces that are spatially detached, we can create non-coexistent trace pairs. Figure 14 shows the $\rho_{max}$ values for such non-coexistent phone traces arbitrarily selected from the Pisa dataset. In the figure, for instance, `1/2-2/1’ refers to the correlation between the second trace of the first campaign (`1/2’) and the first trace of the second campaign (`2/1’). For the five non-coexistent pairs, we observe the same qualitative results. The $\rho_{max}$ remains static except at very low $f_{c}$. In particular, the coefficient at $f_{c}=0.5$ Hz is comparable to that at 5 Hz. False positive decisions are hardly more likely with $f_{c}^{*}=0.5$ Hz than with $f_{c}=5$ Hz, especially when the highest $\rho_{max}$ is much lower than the lowest correlation in the coexistent cases with the same $f_{c}^{*}$ (see Figure 9).

**Figure 15:**
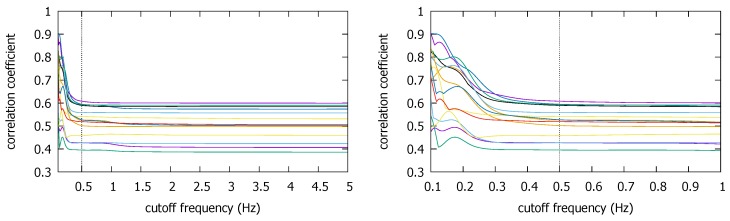
$\rho_{max}$ for non-coexistent trace pairs from the three buildings in the Korea University dataset.

The same observation holds for the Korea University traces, as shown by Figure 15. Here, we match the traces selected from different buildings. The $\rho_{max}$ starts to visibly ascend to large values below 0.5 Hz. However, as long as we stop at the recommended value, there is little concern for a false positive decision. It becomes more clear if we compare the $\rho_{max}$ values in Figure 15 to those that are over 0.9 in coexistent KU trace pairs (Figure 8). Therefore, setting $f_{c}$ at 0.5 Hz (i.e., $f_{s}$ at 1 Hz) for both the Pisa and the KU traces hardly increases the correlation coefficient, hence there is little risk of false positive detections by doing it.

Through the exploration of three independent magnetometer datasets, we commonly observe that removing the high frequencies does *not* significantly change $\rho_{max}$ for the non-coexistent trace pairs unless we suppress $f_{c}$ to a very low value, e.g., below 0.5 Hz. Last but not least, we also observe that $\rho_{max}$ values for non-coexistent conditions do not significantly exceed 0.6 in all our traces. Although we would need more measurement data to validate it, we think this observation will serve as a guide in identifying the desirable decision threshold θ in future work.

### 3.3. Performance of 1 Hz Sampling

Although the LPF has been used to show that it is the low frequencies that have the most relevant signal for the human co-existence detection, we should leverage on the finding to actually lower the original sampling frequency in the first place for the battery saving. Below, we explore what would happen if we did originally sample at 1 Hz. Since the Korea, Pisa, and Illinois traces have all been produced at higher frequencies, we need down-sample (decimate) them to emulate the traces originally produced at 1 Hz. With these down-sampled traces, we first validate our finding that 1 Hz sampling is as good as any higher rate sampling for the magnetometer-based human contact detection. Then, we perform the native 1 Hz sampling in our environment, and compare it with the higher frequency sampling.

#### 3.3.1. Performance of Emulated 1 Hz Sampling

Table 2 shows the result for the coexistent traces in the Illinois dataset. The second column shows the original correlation among these traces with the sampling frequency of 50 Hz. The low-pass filtering with $f_{c}=0.5$ Hz in the third column (i.e., $f_{s}=1$ Hz) produces generally higher correlations, as we saw in Figure 10. The 1 Hz downsampled (decimated) correlations tend to be in between the original and the LPF. We notice that the difference Δ from the original correlation coefficient in 1 Hz sampling is not as significant as to affect the first decimal place.

In the Pisa dataset, we obtain similar results as well (Table 3). For the total of 11 co-existent trajectories, the LPF case again results higher correlation. For the decimated traces, their correlation is comparable to the original.

Table 4 is for the KU dataset. Again, the LPF at $f_{c}=0.5$ Hz returns higher correlation than the original trace. With decimation, the coefficients are also slightly higher, although the difference is smaller. Still, it demonstrates that if we did sample at 1 Hz in the first place, it would be as good as than the original 10 Hz sampling.

In summary, we consistently observe in all three independent datasets that the LPF boots correlation coefficients by eliminating the high frequency components. The emulated 1 Hz sampling through decimation is not as effective, but it generally leads to a higher correlation than the one from the originally high sampling rate. Even if not, the difference is minimal, so it can be used to draw the same conclusion as with the higher rate sampling.

#### 3.3.2. Performance of Native 1 Hz Sampling

Finally, we apply the recommended sampling frequency of 1 Hz to actual trace generation and compare the resulting correlation for the coexistence cases with that of the higher original sampling frequency. Here, we performed another experiment for the same paths in the three buildings in Korea University while collecting the magnetometer traces at native 1 Hz on a different day. The result shown in Table 5 tells us that the variation is slightly larger than the decimated emulation above. It may be because the native 1 Hz sampling data are from a different campaign than the 10 Hz data. Nevertheless, the difference Δ from the original correlation coefficient in the 10 Hz sampling is not as significant as to affect the first decimal place. Therefore, it corroborates our observation that 1 Hz sampling is enough to judge the human coexistence. Moreover, the correlation coefficient for the coexistent condition is very high. Table 6 shows the average $\rho_{max}$ between native 1 Hz sampled traces picked from different buildings (i.e., non-coexistence). As we see, the coefficients are substantially lower than in the coexistent cases in Table 5. Therefore, there is large gap between the coexistent and the non-coexistent trace pairs in between we can pick the decision threshold θ. The gap in the Korea University dataset is larger than the Pisa and the Illinois datasets, possibly because of the differences in traces generation methods as we mentioned in Section 3.1.3. As a clear separation between the coexistent and the non-coexistent cases is desired to set the decision threshold θ, we need further exploration on this issue in future.

Although further corroboration through more exhaustive and independent measurement data is in order, the indoor smartphone magnetometer traces from Korea, Italy, and the U.S. strongly suggest that the sampling rates no higher than 1 Hz are likely to be sufficient for the indoor human coexistence detection purpose. The frequency components above 1 Hz hardly contribute to the relevant signal that affects the coexistence detection, if not interfere with it. This is most likely a unique characteristic of the human contact detection application, and contrasts with other applications such as indoor location and navigation that typically require higher sampling frequencies.

## 4. Discussion

The emergency contact tracing in infectious disease epidemics or the contact monitoring in social studies could use smartphones for detecting encounters between people that may take place anytime, anywhere. Human coexistence monitoring using the smartphone magnetometer traces seems to be only facilitated by paying attention to the low frequency components, in particular those below 0.5 Hz. Reducing the sampling frequency can greatly help suppress the additional battery consumption cost due to the magnetometer sensing. In particular, the 1 Hz sampling we propose in this paper leads to more than two hours longer operating time than 10 Hz sampling on a typical smartphone, not to mention the greater differences it makes against even higher sampling frequencies (e.g., more than five hours and seven hours longer than 50 Hz and 100 Hz sampling, respectively). We believe that it will be crucial for the practicability of the concept of magnetometer-based contact monitoring that should continually run in the background.

The sufficiency of low frequency sampling contrasts to other indoor magnetometer-based applications such as location or navigation, where much higher sampling frequencies ranging from 10 to 200 Hz were used. The difference stems from the appliation requirements. Since the location and navigation may require centimeter-level precision (e.g., for robots), the fine-grained magnetic strength map from the denser measurements in the given indoor environment is desired. However, for human contacts, the required granularity is typically on the order of meters [11]. We believe this translates to the more lenient sampling frequency requirement in the human contact monitoring.

From the three datasets, we estimate that the decision threshold θ= 0.7 would work to distinguish coexistence from non-coexistence. However, more of the publicly available magnetometer traces in the future will give us a chance to review the relevance of the threshold value. We also assumed that the coexistence should last longer than 20 s. In case shorter encounters should be monitored as well, we will have to evaluate the impact of the window size over which the correlation is computed. Finally, the `same-place-different-time (SPDT)’ disease transmissions [31] (or `co-location’ [30]) will be an interesting extension of the current work. We believe such contact types will be detected by coping with time gaps and distortions stemming from moving speed differences and detours. However, we leave it for a future work.

In this work, we relied on the Android getRotationMatrix() method to cope with the phone attitude changes. However, an alternative, purely magnetometer-based approach is to exploit the fundamental property of the magnetic disturbances on two close devices [32]. Genovese et al. showed that ambient magnetic disturbances affect either more heavily, but the heading errors on the devices are similar in magnitude. We will look into this solution in our future work. Another issue is the coexistence detection range of the proposed method. Since the strength of a magnetic field is inversely proportional to the square of the distance from the source, our method has a limit on the detection range to a few meters, as shown by our early work [8]. For infectious disease transmission, this is acceptable. However, for other applications that require a larger range, an extension of the proposed mechanism is certainly in order. We will explore on this aspect in future work. Finally, as to the shoulder-to-shoulder formation used for our experiments, one might wonder how other formations affect our method. Our early pilot study shows that it can be made to work on other bundle formations such as a column or an oblique line with small time shifts in correlation computation [33].

## 5. Conclusions

Smartphone magnetometers can provide useful information for human coexistence detection. It is precise enough to detect a contact within a few meters, does not require infrastructure, does not expose identity or location. However, recent works based on magnetometers do not address the practicability aspect of the idea, namely the battery consumption due to continuous operation. In order to monitor the potentially infectious contacts that may take place anytime, the magnetometer sensing should be continuously performed in the background. This can put a strain on the smartphone’s energy budget, which may significantly undermine the practicability of the magnetometer-based contact monitoring application. This paper investigates the appropriate frequency of sensing that minimizes the energy cost while not losing the efficacy of magnetometer-based coexistence detection. By investigating the independently produced indoor magnetometer traces from locales in three different countries, we find that the frequency components above 0.5 Hz hardly contribute to the signal useful for the coexistence detection. It directly implies that sampling the magnetic field strength at 1 Hz is enough for the application. In terms of the battery drain time, the low sampling rate can increase the time-to-drain of a 2800 mAh pack by more than two hours (compared with 10 Hz sampling), more than seven hours (compared with 100 Hz sampling), and almost 11 h (compared with 200 Hz sampling). With other tasks running simultaneously, the energy saving aspect will become only more critical for the practical application of the magnetometers in the continuous human coexistence detection.

## Figures and Tables

**Table 1:** Energy consumption due to magnetometer sensing at various frequencies on Galaxy S5.

Sampling Frequency	Average Current Draw*I* (mA)	Expected Time-to-Drain (TTD)	$\Delta_{\mathrm{TTD}}$
baseline	118.50	23 h 37 min	0
0.5 Hz	119.40	23 h 27 min	−0 h 10 min
1 Hz	122.24	22 h 54 min	−0 h 43 min
2 Hz	123.59	22 h 39 min	−0 h 58 min
5 Hz	127.95	21 h 52 min	−1 h 45 min
10 Hz [8,12]	134.76	20 h 46 min	−2 h 51 min
25 Hz [13,14]	142.54	19 h 39 min	−3 h 58 min
49.65 Hz [10]	153.62	18 h 13 min	−5 h 24 min
50 Hz [15]	156.72	17 h 52 min	−5 h 45 min
100 Hz [16,17]	178.61	15 h 40 min	−7 h 57 min
200 Hz [18]	231.66	12 h 05 min	−11 h 32 min

**Table 2:** Average $\rho_{max}$ between the coexistent Illinois traces before and after LPF/decimation.

Building	50 Hz	1 Hz (LPF)	1 Hz (Decimated)	Δ
CSL	0.798	0.822	0.813	+0.016
Loomis	0.912	0.964	0.941	+0.029
Talbot	0.875	0.912	0.887	+0.012

**Table 3:** Average $\rho_{max}$ in the coexistent Pisa traces before and after LPF/decimation.

Campaign	10 Hz	1 Hz (LPF)	1 Hz (Decimated)	Δ
1	0.923	0.935	0.921	−0.002
2	0.930	0.945	0.940	+0.009

**Table 4:** Average $\rho_{max}$ for the coexistent KU traces before and after LPF/decimation.

Building	10 Hz	1 Hz (LPF)	1 Hz (Decimated)	Δ
ECB	0.946	0.959	0.947	+0.001
CTH	0.963	0.982	0.974	+0.011
CSQ	0.951	0.962	0.951	+0.000

**Table 5:** Average $\rho_{max}$ for the native 1 Hz sampled traces.

Building	10 Hz	1 Hz (Real)	Δ
ECB	0.946	0.984	+0.038
CTH	0.963	0.933	−0.030
CSQ	0.969	0.948	−0.021

**Table 6:** Average $\rho_{max}$ for the non-coexistent 1 Hz sampled traces.

Building	1 Hz (Real)
<CSQ,CTH>	0.440
<CSQ,ECB>	0.556
<CTH,ECB>	0.425

## Supplementary Materials

The Korea University traces are available online at https://widen.korea.ac.kr/wiki/index.php/KU-indoor-magnetometer-traces.
